# Multi-Community Cardiopulmonary Resuscitation Education by Medical Students

**DOI:** 10.7759/cureus.8647

**Published:** 2020-06-15

**Authors:** Kenton L Anderson, Kian Niknam, Larry Laufman, Stefanie S Sebok-Syer, Sara Andrabi

**Affiliations:** 1 Emergency Medicine, Stanford University School of Medicine, Palo Alto, USA; 2 Medicine, Baylor College of Medicine, Houston, USA; 3 Emergency Medicine, Stanford University, Palo Alto, USA; 4 Emergency Medicine, Baylor College of Medicine, Houston, USA

**Keywords:** cardiopulmonary resuscitation, education, medical students, community participation

## Abstract

Introduction

One purpose of the hands-only cardiopulmonary resuscitation (HOCPR) program is to simplify CPR instruction to encourage more bystanders to take action during cardiac arrest. Although the program has been successfully implemented in traditional classroom settings, the utility of large-scale training events has not been well-explored. We hypothesized that CPR knowledge and comfort levels would increase through a large-scale, multi-community HOCPR training event. We also explored what effect this training event had on perceived barriers to bystander-performed CPR.

Methods

A convenience sample participated in HOCPR training on a single day across 10 Texas cities. A sub-sample completed training questionnaires, including a five-item CPR pre- and post-test. A follow-up questionnaire was conducted two years after the event. The primary outcome of interest was the difference in cardiopulmonary resuscitation (CPR) knowledge and comfort level between pre- and post-event questionnaires. Demographic contributions were also assessed.

Results

A total of 4,253 participants were trained, 1,416 were enrolled upon submitting matching pre- and post-event questionnaires, and 101 (14%) submitted follow-up questionnaires. Mean knowledge scores increased from pre-training (2.7 ± 1.6 standard deviation (SD)) to post-training (4.7 ± 0.76 SD) (p < 0.001). Follow-up test scores (3.8 ± 1.1 SD) remained higher than pre-test scores (p < 0.001). Comfort with HOCPR increased from 59% (95% confidence interval (CI) 56 - 61) to 96% (95% CI 95 - 97). Pre- and post-knowledge scores differed significantly by education level (p < 0.001), ethnicity (p < 0.001), and income (p < 0.001). Education contributed significantly to comfort at both pre- (p = 0.015) and post-training (p = 0.026), but ethnicity and income did not. Before training, the most common barrier to performing CPR was lack of knowledge 59% (95% CI 55 - 62); after training, the most common barrier was fear of causing harm 34% (95% CI 29 - 40).

Conclusions

This study demonstrated that medical students were successfully able to conduct large-scale HOCPR training that improved CPR knowledge and comfort levels among participants across multiple metropolitan areas. Knowledge retention remained higher at two-years for participants of a follow-up questionnaire. Medical students can use the experiences from this training event as a template to organize similar large-scale training events in the future.

## Introduction

Cardiac arrest is one of the leading causes of death among adults over the age of 40; hence, even small incremental improvements in survival can translate into thousands of lives saved each year [1-3]. Bystander cardiopulmonary resuscitation (CPR) is a key factor in the Chain of Survival. Although at least half of out-of-hospital cardiac arrests (OHCA) are witnessed and bystander-initiated CPR can substantially increase the likelihood of survival, the rate of bystander CPR remains low in most countries [1, 3, 4-8]. In recent years, there has been an increased effort to teach bystanders hands-only CPR (HOCPR) since it has similar survival rates as conventional CPR, is easier to teach, remember, and perform, and was designed to overcome potential barriers to bystander performance of CPR, such as panic and reluctance to perform mouth-to-mouth ventilations [9-10].

HOCPR training has been implemented through traditional classroom programs. However, the rate of bystander CPR in many communities and demographic groups remains very low [11]. Numerous strategies have been implemented to reach a larger number of lay rescuers, including video self-instruction and media [12-13]. In one instance, a single large-scale training session was used to train over 5,000 bystanders about conventional CPR in Singapore, but the utility of large-scale training sessions using HOCPR has not yet been well-explored [14]. 

In this study, we aimed to create a single-day state-wide HOCPR training event that was organized and conducted by medical students. The goal of our study was to assess the feasibility of using a large-scale HOCPR training event, conducted by medical students, to increase participant CPR knowledge and their comfort level performing HOCPR. We also explored what effect this training event had on perceived barriers to bystander-performed CPR.

## Materials and methods

Study design and setting

**Figure 1:**
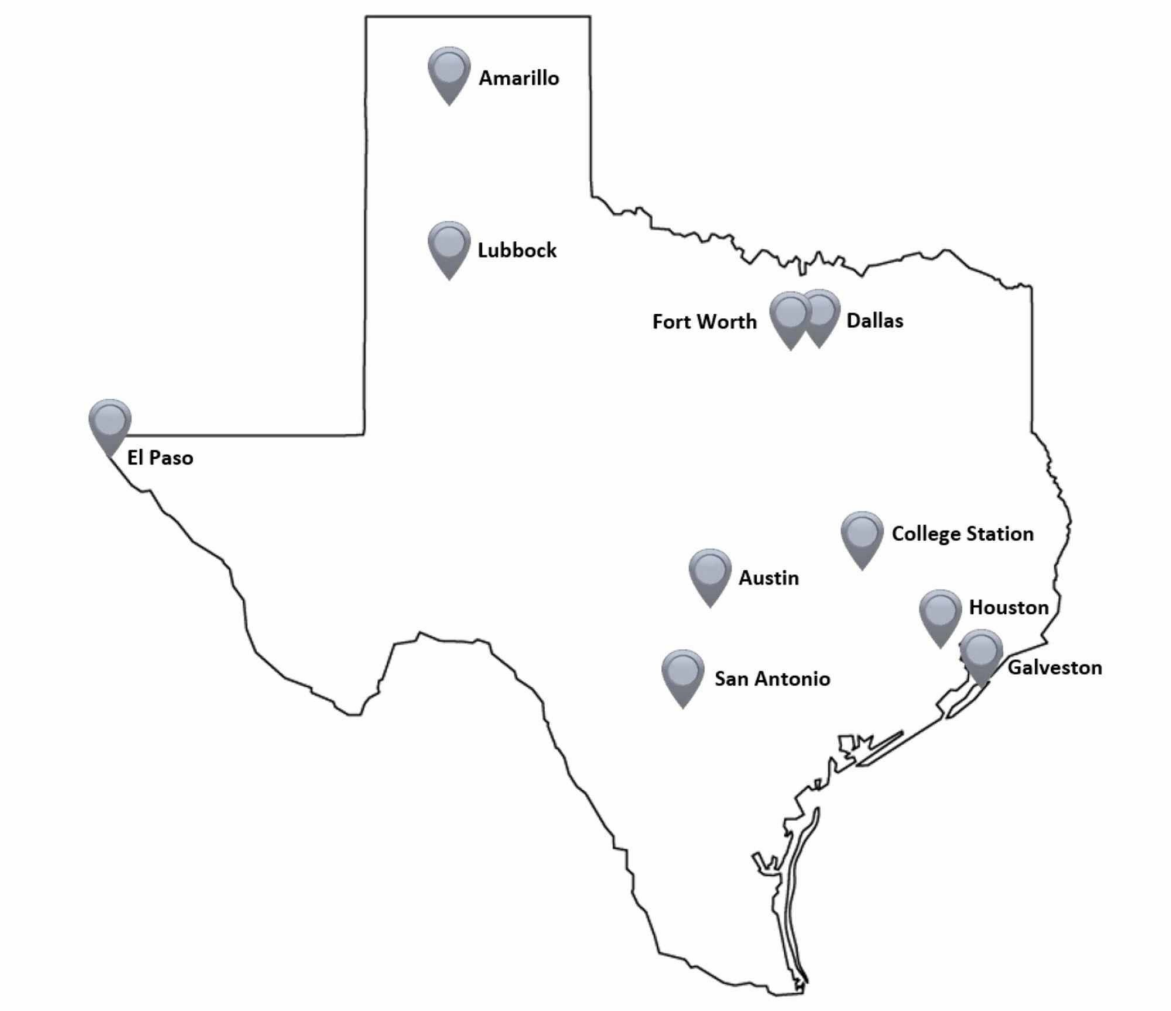
Training cities

The Texas Two-Step CPR training event was organized and conducted by medical students on a single day (February 6, 2016) at 53 public locations in 10 cities across the state of Texas (Figure 1). Public locations for the event included college campuses, museums, recreation centers, retail centers, healthcare clinics, religious centers, and a theater. The event Board of Directors (BOD) included six medical students who organized operations, communications, public relations, and media, as well as event registration and research. The BOD recruited medical student volunteers from each of the nine Texas medical schools and instituted a board of four students from each institution to direct 1) volunteers, 2) locations, 3) equipment, and 4) outreach at their respective institutions. 

To evaluate the effect of the event, a convenience sample of participants attending the first annual Texas Two-Step CPR training event completed a pre- and post-event questionnaire of CPR knowledge and comfort level. All adult participants (age ≥ 18 years) were eligible for inclusion; the initial intent was to include all event participants, but several event site leaders decided not to distribute the questionnaires because doing so disrupted the flow of participants through the educational event. Participants were excluded if they were unable to complete the written questionnaire in English or had a physical disability that precluded them from participating in the training event. The study was approved by the Institutional Review Board with a waiver of documentation of informed consent; reporting follows the Strengthening the Reporting of Observational Studies in Epidemiology (STROBE) statement for observational studies [15].

Intervention

Following recruitment and informed consent, participants completed a pre-training registration form, including demographic information, prior CPR training, follow-up contact information, and CPR comfort level, as well as the HOCPR pre-test developed by the American Heart Association (AHA) (Appendix 1-2) [16]. Participants subsequently watched a five-minute scripted demonstration of HOCPR in either English or Spanish performed by student volunteers trained in HOCPR. Training also introduced the Good Samaritan Law to alleviate concerns regarding legal liability when performing CPR in public [17]. Participants were subsequently allowed a five to 10 minute assisted practice session with feedback from the medical student proctors until they were able to independently perform HOCPR correctly, including a simulated call to emergency medical services (911) and closed-chest compressions on mannequins at the appropriate rate and depth. After completion of the training, participants completed a post-event questionnaire, including CPR comfort level and the AHA-developed HOCPR post-test (Appendix 3) [16]. 

Follow-up

For participants who agreed to participate in the follow-up survey, a questionnaire with the same CPR comfort level questions and HOCPR post-test (Appendix 3) were sent to participants to be completed 23-24 months following the Texas Two-Step CPR training event [16]. The follow-up questionnaire included two additional questions asking whether participants had either shared or used the CPR knowledge they gained (Appendix 4).

Outcomes

The purpose of this study was to determine whether it was feasible for medical students to organize a large-scale multi-community HOCPR training event. Similar to prior CPR training studies, to determine if this event might have any potential public health benefit, the primary outcomes of interest were the difference in CPR knowledge and comfort level between pre- and post-event surveys [18]. Demographic contributions to pre- and post-event knowledge and comfort level, as well as two-year follow-up knowledge and comfort level, were also assessed. Participant responses to open-ended questions are listed in Appendix 5.

Analysis

Paired sample t-tests and Wilcoxon rank sign tests were used, as appropriate, to compare pre-, post-, and follow-up tests. Additionally, we used descriptive statistics to examine the distribution of primary outcomes and variables around 95% confidence intervals. Spearman’s correlation was used to assess relationships between participant demographics and test performance. Analysis of variance and correlation using both parametric and non-parametric statistics were used as appropriate. Analyses were run using Stata 15.1/SE for Windows (StataCorp, LP College Station, TX).

## Results

Study participants

**Figure 2:**
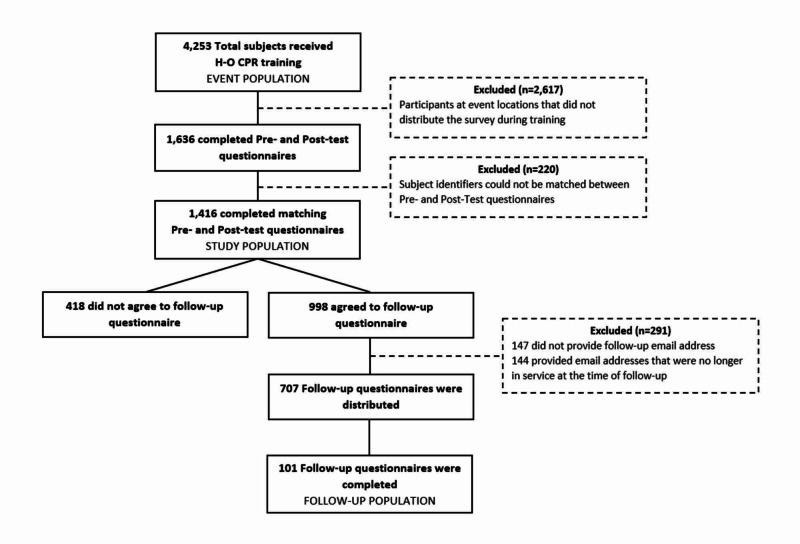
Study population HOCPR: hands-only cardiopulmonary resuscitation

Four thousand two hundred and fifty-three participants received HOCPR training during the single-day Texas Two-Step CPR training event. Nineteen of the 53 training locations distributed the pre- and post-test questionnaires so 1,636 questionnaires (38% of all participants) were distributed (Figure 2). Of the completed questionnaires, an additional 220 had subject identifiers that could not be matched between the pre- and post-test; thus, 1,416 (87%) of the pre-and post-tests were available for analysis. Nine hundred and ninety-eight (70%) of the subjects that submitted matching questionnaires at the event expressed willingness to participate in a follow-up survey and 707 (49%) provided usable electronic mail addresses for follow-up. One hundred and one (14%) of the subjects that provided usable contact information submitted follow-up questionnaires. Demographics are listed in Table 1. The training-day and follow-up samples did not differ in terms of prior CPR training, pre-test, or post-test knowledge scores; however, compared with the training-day sample, the follow-up sample was more comfortable performing CPR (p < 0.001), was about five years older (p = 0.003), and had a higher level of education (p = 0.015). 

**Table 1:** Demographic Background of Respondents *See Appendix 5 for responses to ‘Other.’ Totals vary due to non-responses. CI: confidence interval; CPR: cardiopulmonary resuscitation; GED: general educational development; IQR: interquartile range; N: number

Variable		Training day		Follow-up
	N	Median (IQR) or % (95% CI)	N	Median (IQR) or % (95% CI)
Age, years	1,445	24 (19 - 38)	101	30 (20 - 47)
Gender				
Female	703	54 (51 - 57)	60	61 (50 - 70)
Male	603	46 (43 - 49)	39	39 (30 - 50)
Total	1,306		99	
Ethnic background				
White	508	39 (36 - 41)	48	48 (38 - 58)
Hispanic/Latino	414	32 (29 - 34)	24	24 (16 - 34)
Asian	162	12 (11 - 14)	10	10 (4.9 - 18)
Black/African American	117	9.0 (7.4 - 11)	11	11 (5.6 - 19)
Other/Mixed	52	3.9 (3.0 - 5.1)	2	2.0 (0.2 - 7.0)
Prefer not to answer	63	4.8 (3.7 - 6.1)	5	5.0 (1.6 - 11)
Total	1,316		100	
Annual household income				
< $25,000	169	13 (12 - 15)	17	18 (11 - 27)
$25,000 - $49,999	178	14 (12 - 16)	13	14 (7.4 - 22)
$50,000 - $74,999	154	12 (11 - 14)	13	14 (7.4 - 22)
$75,000 - $99,999	124	9.8 (8.3 - 12)	5	5.2 (1.7 - 12)
$100,000 - $149,999	138	11 (9.3 - 13)	16	17 (9.8 - 26)
$150,000 or more	123	9.8 (8.2 - 12)	12	13 (6.6 - 21)
Prefer not to answer	374	30 (27 - 32)	20	21 (13 - 30)
Total	1,260		96	
Highest education level				
Did not complete any schooling	35	2.8 (2.0 - 3.9)	0	0.0
Did not graduate from high school	150	12 (10 - 14)	6	6.1 (2.3 - 13)
High school degree or equivalent (e.g., GED)	168	13 (12 - 15)	11	11.1 (5.7 - 19)
Some college but no degree	357	29 (26 - 31)	23	23 (15 - 33)
Associate or technical degree	59	4.7 (3.6 - 6.0)	9	9.1 (4.2 - 17)
Bachelor’s degree	306	24 (22 - 27)	32	32 (23 - 43)
Master’s degree	116	9.3 (7.7 - 11)	12	12.1 (6.4 - 20)
Professional or Doctorate degree	61	4.9 (3.7 - 6.2)	6	6.1 (2.3 - 13)
Total	1,252		99	
Previous CPR Training				
Yes	568	46 (43 - 48)	47	50 (40 - 61)
No	681	55 (52 - 57)	47	50 (40 - 61)
Total	1,249		94	
Reasons to learn CPR				
To learn how to help someone in need	1016	62 (60 - 64)	87	67 (59 - 75)
Work requirement	184	11 (9.7 - 13)	10	7.7 (3.1 - 12)
None of the above	64	4.0 (2.8 - 4.9)	1	0.8 (0.7 - 2.3)
Personally know someone with heart disease	133	8.1 (6.8 - 9.5)	14	11 (5.4 - 16)
To teach others CPR	152	9.3 (7.9 - 11)	13	10 (4.8 - 15)
Other*	87	5.3 (4.2 - 6.4)	5	3.8 (0.5 - 7.2)
Total	1,636		130	

CPR knowledge

Mean CPR knowledge scores improved between pre-and post-tests on the day of the training event (Table 2). Although the mean follow-up test scores decreased from the post-test scores, the follow-up scores remained higher than the pre-test scores. 

**Table 2:** Mean Pre-Test, Post-Test, and Follow-up Test Scores for Five CPR Knowledge Questions CPR: cardiopulmonary resuscitation; N: number; SD: standard deviation

Survey	N	Mean ± SD	p-value
Training Pre-test	1,414	2.7 ± 1.6	< 0.001
Training Post-test	1,414	4.7 ± 0.76	
Training Post-test	100	4.8 ± 0.47	< 0.001
Follow-up	100	3.8 ± 1.1	
Training Pre-test	98	2.9 ± 1.4	< 0.001
Follow-up	98	3.8 ± 1.1	

Higher education levels correlated significantly with higher pre- (r = 0.266, p < 0.001) and post-test (r = 0.147, p < 0.001) scores, but not follow-up test scores (r = 0.0384, p = 0.707). Those whose highest level of education was a bachelor’s degree performed best on both pre- and post-tests (3.3 ± 1.4 and 4.8 ± 0.4, respectively), while those who did not complete any schooling performed the worst on both pre- and post-tests (1.5 ± 1.5 and 4.4 ± 1.2, respectively). During the follow-up, those whose highest level of education was an associate or technical degree performed the best on the test (4.1 ± 0.6), while those whose highest level of education was a high school degree or equivalent performed the worst (3.4 ± 1.6).

Similarly, there was a significant correlation between higher family incomes and higher pre- (r = 0.267, p < 0.001) and post-test scores (r = 0.170, p = < 0.001), but this correlation was not significant in follow-up scores (r = 0.002, p = 0.986). It was found that those who had a family income of < $25,000 performed worst on both pre- (2.0 ± 1.7) and post-tests (4.5 ± 0.9), but those who had an income of ≥ $150,000 performed the best on both tests (pre: 3.3 ± 1.1, post: 4.8 ± 0.5). On the follow-up test, those whose family income was 75,000 - 99,999 performed best on average (4.8 ± 0.5) while those who made 25,000 - 49,999 performed worst on average (3.5 ± 1.1).

There was also a significant difference between ethnic background and pre- and post-test scores (p < 0.001) with White/Caucasian participants receiving the highest scores on average (pre: 3.2 ± 1.3, post: 4.8 ± 0.5) and Other/Mixed receiving the lowest scores on average (pre: 2.0 ± 1.7, post: 4.4 ± 1.2). There was not a significant relationship between ethnic background and follow-up scores (p = 0.948) with Other/Mixed performing the best on average (4.0 ± 1.4) and Hispanic/Latino performing the worst on average (3.6 ± 1.3).

CPR comfort level

Reported CPR comfort level improved between pre- and post-event questionnaires (p < 0.001) (Table 3). Although there was a trend toward decreased comfort levels between the post-test and the follow-up (p = 0.220), follow-up comfort remained higher than pre-test comfort (p = 0.029).

**Table 3:** Responses to Pre-Test, Post-Test, and Follow-up Questions About the CPR Training *See Appendix 5 for responses to ‘Other’ CI: confidence interval; CPR: cardiopulmonary resuscitation

Question	Pre-Test	Post-Test	Follow-Up
	% (95% CI)	% (95% CI)	% (95% CI)
Would you feel comfortable performing Hands-Only CPR if someone had a cardiac arrest?			
Yes	59 (56 - 61)	96 (95 - 97)	78 (69 - 86)
No	41 (39 - 44)	4.1 (3.1 - 5.2)	22 (14 - 31)
If you would NOT feel comfortable performing Hands-Only CPR now, please explain why:			
I don’t feel I know how to correctly perform CPR	59 (55 - 62)	19 (15 - 24)	31 (15 - 51)
I may be legally liable if I get involved	6.6 (5.0 - 8.6)	18 (14 - 23)	31 (15 - 51)
I may hurt someone or do CPR wrong if I get involved	19 (16 - 22)	34 (29 - 40)	14 (3.9 - 32)
I am afraid of getting a disease	2.3 (1.4 - 3.6)	2.2 (0.8 - 4.7)	2.9 (1.0 - 8.3)
Other*	14 (11 - 16)	27 (22 - 33)	0.0 (0.0 - 3.6)
Would you recommend this CPR training to a friend of a family member?			
Yes	-	99 (99 - 100)	99 (95 - 100)
No	-	0.7 (0.3 - 1.4)	1.0 (0.0 - 5.5)
Have you told anyone about what you learned in your CPR training?			
Yes	-	-	53 (42 - 63)
No	-	-	47 (37 - 58)
Have you used what you learned about CPR since the training in February 2016?			
Yes	-	-	4.0 (1.1 - 10)
No	-	-	96 (90 - 99)

Education contributed significantly to comfort at both pre- (p = 0.015) and post-training (p = 0.026) but not for follow-ups (p = 0.062). Comfort did not differ significantly by family income nor ethnicity in any of the tests. On average, those who felt comfortable during the pre-test scored 0.7 higher (95% CI: 0.5 - 0.9, p-value < 0.001) than those who felt uncomfortable. During the post-test, those who felt comfortable scored, on average, 0.9 higher (95% CI: 0.7 - 1.1, p-value < 0.001). Similarly, during the follow-up test, those who felt comfortable scored 0.6 higher on average (95% CI: 0.2 - 1.2, p-value = 0.010).

Perceived barriers

The most common pre-training reason for not being comfortable with CPR was not knowing how to correctly perform CPR (Table 3). Post-training, the most common reason was a concern for hurting someone. However, during follow-up, the most common reason was a tie between not knowing how to correctly perform CPR and being legally liable if they got involved.

## Discussion

In our study, we found that medical students were able to successfully organize and conduct a large-scale HOCPR training event with improvement in participant CPR knowledge and comfort-level performing HOCPR. We report a 40% increase in CPR knowledge based on the AHA HOCPR pre- and post-tests, as well as a 37% increase in the number of participants that felt comfortable performing HOCPR after the single-day training event. Although the follow-up response rate was low, CPR knowledge and comfort were retained somewhat even two years later.

The Texas Two-Step CPR training event represents a new method of educating bystanders that does not require any prior CPR instructor experience. Although the organizers of this event were medical students with some experience with CPR, none had prior experience conducting CPR education sessions. Previous studies have demonstrated that school teachers with no prior CPR education experience were also able to conduct similar HOCPR training in a public-school setting [19]. Although HOCPR training methods vary substantially, “ideal CPR education sessions should be accessible for all people and result in increased knowledge and comfort among participants" [19-20]. In this regard, the Texas Two-Step event was tremendously successful. The ethnic background of our study population (Table 1) closely reflects the demographics of the state of Texas which suggests that the training was indeed accessible and used by a representative cross-section of the targeted population [21]. Subsequently, all participants experienced an increase in CPR knowledge and comfort. Due to the ability of this initial training event to teach such a large number of bystanders in such a short period of time, the Texas Two-Step CPR training event has become an annual program that has expanded to 10 states and has trained more than 18,500 participants in HOCPR since inception [22].

In this study, there was a high proportion of participants who expressed comfort performing CPR after the training. In the post-test survey, 96% of participants expressed comfort performing HOCPR; this is higher than previous HOCPR studies [19, 23]. The reason for this discrepancy is likely multifactorial. However, this study demonstrated that the education level contributed to the comfort level, suggesting that participants with lower education levels may benefit from an emphasis on the factors that contribute to discomfort. Fortunately, at least one of the participants was confident enough to use their CPR skills to perform chest compressions during a cardiac arrest case (Appendix 5).

Our study also demonstrated a relationship between higher education and income with higher pre- and post-test scores. Although we did not specifically look for a correlation between education and income levels, this relationship is well-known [24]. Higher pre-test scores suggest that participants with higher education or income either had preexisting CPR knowledge, were better test-takers, or both. Lower education and income have been associated with less exposure to CPR training which is likely the major contributing factor in this study [25]. The relationship between education and higher test scores did not hold true for the follow-up survey which suggests that the questions were sufficiently simple enough that the test-taking strategy had less of an influence, and the exposure to CPR concepts during this training event may have equalized the disparity due to education or income over the long-term. Further analysis would be required to verify this premise. Nonetheless, these findings are encouraging as they suggest that even brief exposure to CPR concepts may have longstanding benefits.

Although bystander CPR increases survival and improves health outcomes from cardiac arrest, less than one-half of out-of-hospital cardiac arrest victims receive bystander CPR [1, 11, 26]. Only a few prior studies have focused on the potential barriers to bystander CPR [27-28]. However, our study echoes previous work suggesting that bystanders without CPR training are less willing to attempt CPR due to their lack of CPR knowledge or experience. After the training event, our study found that the most common perceived barrier became a concern for causing harm which is a barrier that has also been expressed in prior work [27-28]. In contrast to some previous studies, few of our participants were concerned about contracting a disease from victims; this is likely due to the nature of HOCPR which eliminates mouth-to-mouth contact [27-28]. Although HOCPR overcomes some of the barriers to bystander CPR, further work needs to be done to overcome commonly reported barriers - some of which are simply misperceptions among the general public. Large-scale, no-cost training events, such as the one we held in this study, may be one way to rapidly increase CPR knowledge and experience among large numbers of bystanders. These training events could also be used as a platform to address some of the common misperceptions which continue to be barriers to bystander-performed CPR.

Limitations

Our study had some limitations. First, this was a convenience sample of participants during a single day training event and was subject to selection bias. Many of the training location leaders did not distribute the questionnaires because they thought it disrupted the flow of the training session. However, the demographics of our training day population reflect the demographics of the state of Texas which suggests that the training session was successful in reaching a representative subset of the target population (Table 1) [21]. This is encouraging since the African American and Hispanic ethnic groups traditionally do not learn CPR or perform bystander CPR at the rate of other ethnic groups [29-30]. Our study demonstrates that community outreach was able to successfully target these hard to reach groups as well. In fact, the HOCPR training was held in both English and Spanish at some of the sites. However, the questionnaire was not translated into Spanish, so our results may under-represent the Hispanic population who participated in the event. Nonetheless, participants of this training event self-selected rather than being randomly chosen; those who participated in the training may have been more likely to consider providing CPR at baseline. Additionally, there is the potential for a Hawthorne effect in the follow-up survey - participants may have looked up the answers if they wanted a higher score. Second, the results for the follow-up sample may not be representative of the original training-day sample. As mentioned in the results section, the two samples do not differ in terms of prior CPR training, pre-test, or post-test knowledge scores. However, compared with the original sample, the follow-up sample was more comfortable performing CPR, was older, and more educated. These differences may introduce potential selection bias, and the results should be considered accordingly. Lastly, the AHA CPR guidelines changed in October 2015, just a few months before the Texas Two-Step CPR training day. The online pre- and post-test changed in January after materials for the training day had been printed. The questions did not change, but the answer for the correct number of chest compressions in a one-minute period has changed from “at least 100” to “100 - 120.” It is unlikely that this change made any difference since the answers are almost identical, except for the new upper limit of 120. Additionally, in the online follow-up, we were unable to test manual skills, including compression rate, depth, and lean. During the training event, all subjects demonstrated these manual skills, but the format of the follow-up did not allow the assessment of these skills.

## Conclusions

In summary, medical students were able to successfully organize and conduct a large-scale hands-only CPR training event that improved participant CPR knowledge and comfort level. Although there was some decrease in both knowledge and comfort after the event, both remained higher than the pre-test baseline at a two-year follow-up. Our study also identified perceived barriers to bystander-performed CPR change with training and time after training. Medical students can use the experiences from this training event as a template to organize similar large-scale training events in the future.

## Figures and Tables

**Figure 3:**
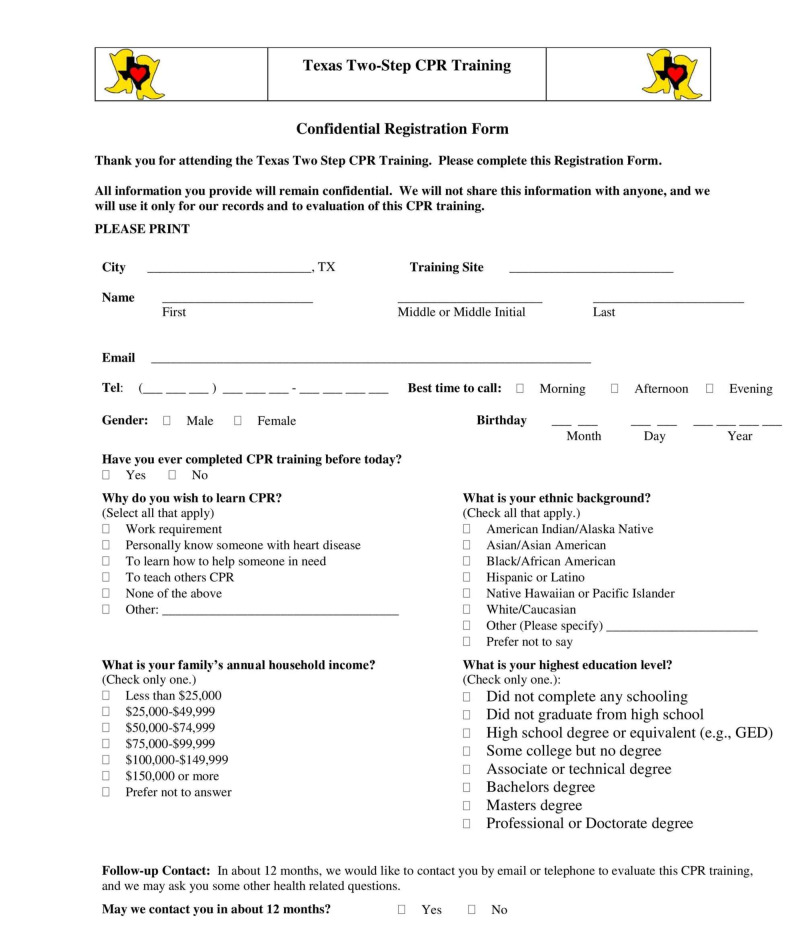
Appendix 1: Registration form

**Figure 4:**
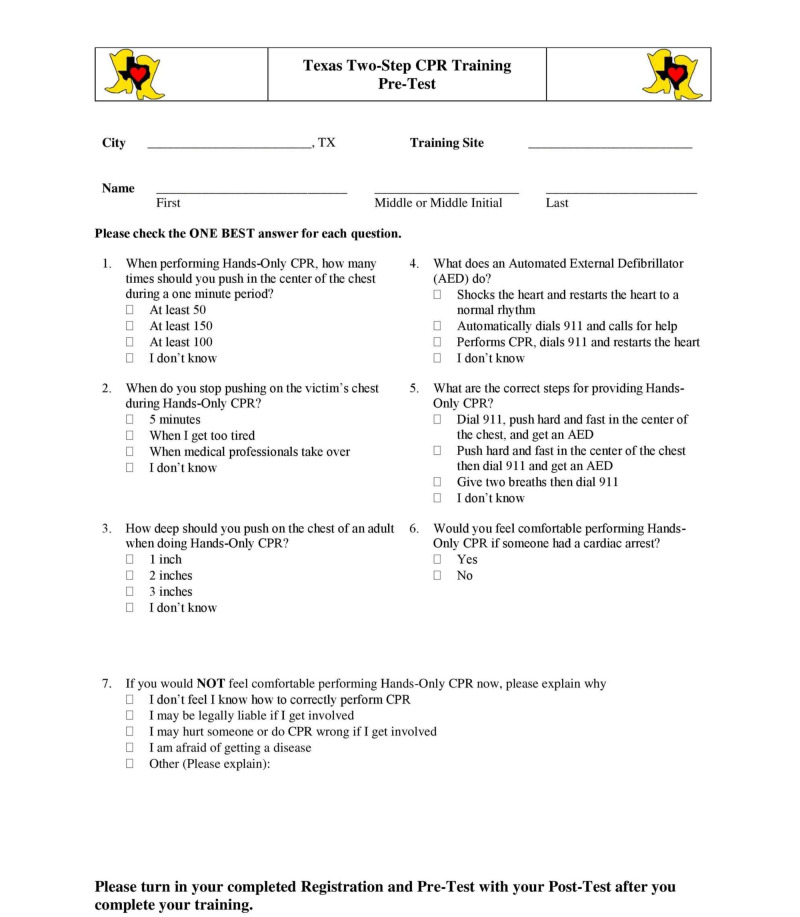
Appendix 2: Pre-test

**Figure 5:**
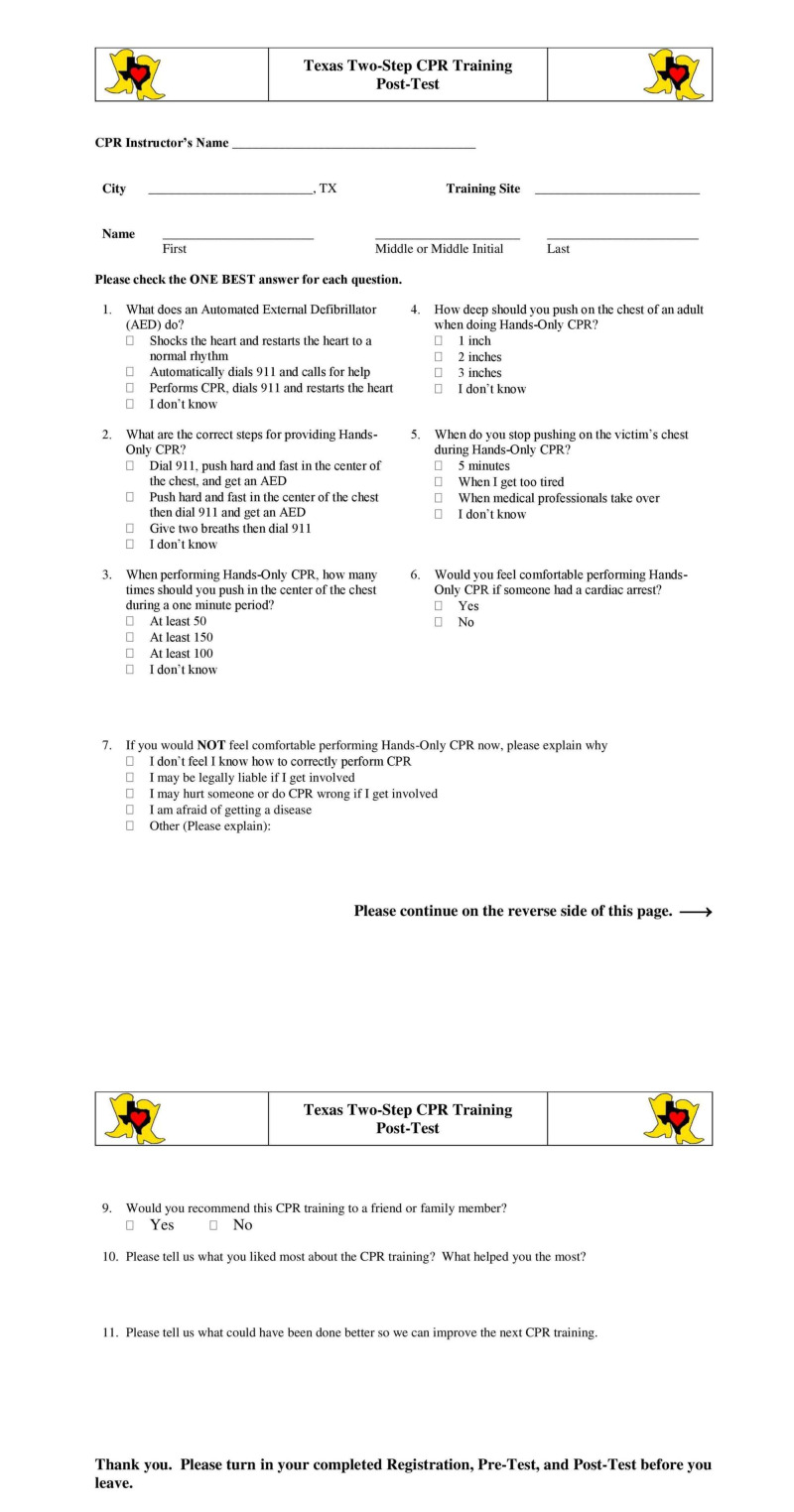
Appendix 3: Post-test

**Figure 6:**
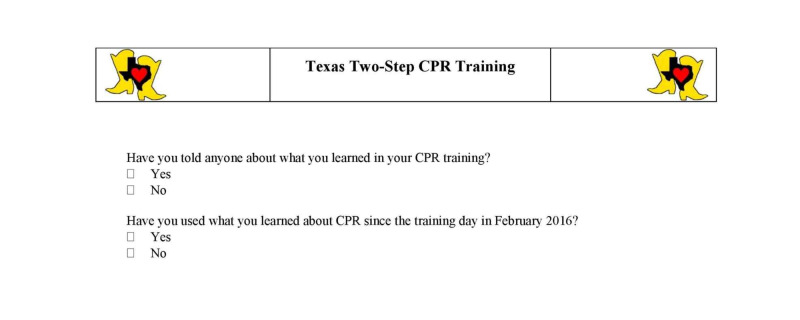
Appendix 4: Follow-up confidence questions

**Figure 7:**
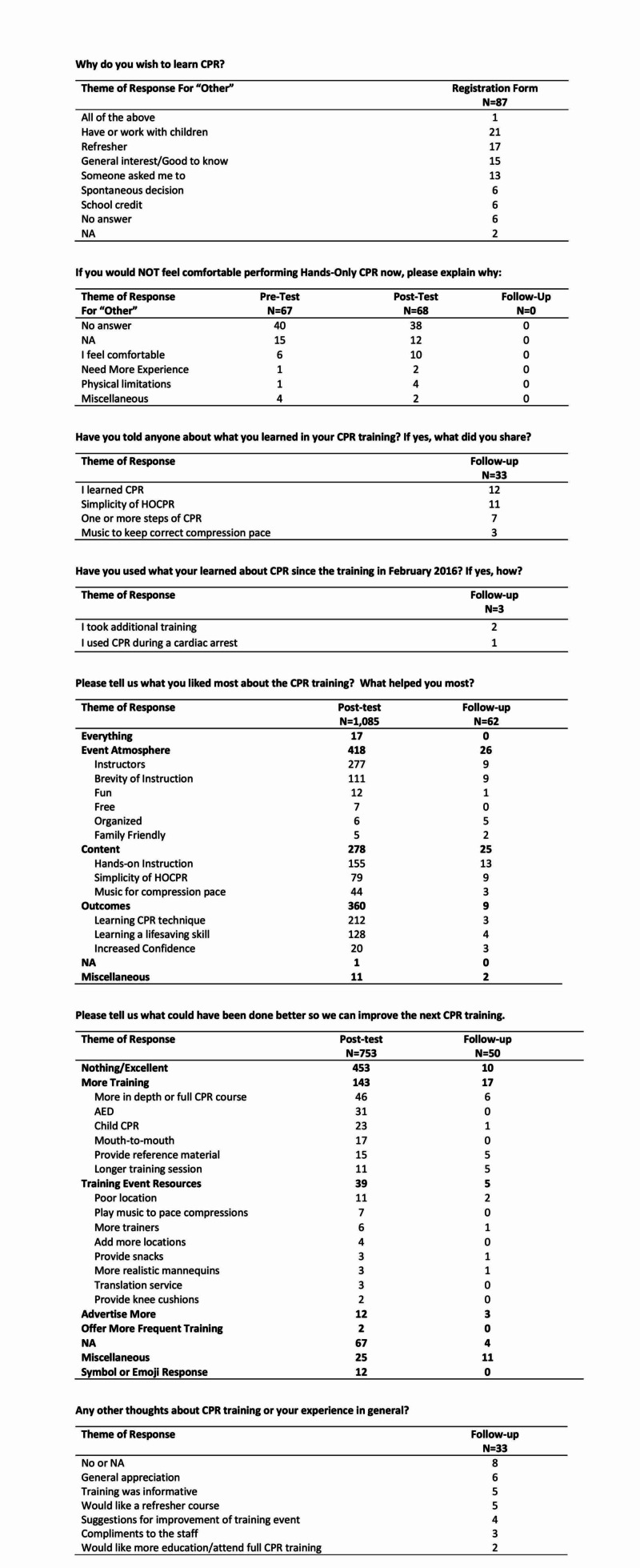
Appendix 5: Tables for open-ended questions
